# Anterior scleritis with IgG4 lymphoplasmacytic infiltration: a case report

**DOI:** 10.1186/s12348-025-00516-3

**Published:** 2025-08-16

**Authors:** Poojitha Balakrishnan, Matthew G. Vicinanzo, John P. Luckett, Tom Winokur, Ryan S. Weldon, Russell W. Read

**Affiliations:** 1 Department of Ophthalmology and Visual Sciences, University of Alabama School of Medicine, 1720 University Boulevard, Suite 302, Birmingham, AL 35233 USA; 2 Alabama Ophthalmology Associates, Birmingham, AL USA; 3 Department of Pathology and Anatomic Pathology, University of Alabama School of Medicine, Birmingham, AL USA; 4 Rheumatology Associates, Birmingham, AL USA; 5 Retina Consultants of Alabama, Birmingham, AL USA; 6 Department of Ophthalmology, Birmingham VA Health Care System, Birmingham, AL USA

**Keywords:** IgG4-related disease, Anterior scleritis, Lymphoplasmacytic inflammatory disease

## Abstract

**Purpose:**

To report a case of chronic unilateral nodular anterior scleritis as a rare, isolated presentation of IgG4-related ophthalmic disease.

**Case:**

A fifty-two-year-old patient was evaluated for painful, nodular scleral injection of the right eye, diagnosed as nodular anterior scleritis. There was only a partial response to topical corticosteroid and systemic immunomodulatory therapy. This, in combination with a sharp delineation between normal and abnormal sclera lead to the decision to perform a diagnostic biopsy of the lesion. Histopathology showed an IgG4 lymphoplasmacytic infiltration of the scleral tissue.

**Conclusions:**

There are few documented reports of scleritis as the presenting manifestation of IgG4-related disease. IgG4-related disease is an increasingly recognized etiology to be considered in evaluating and managing scleritis.

## Introduction

Scleritis is a vision-threatening inflammatory condition that is characterized by severe pain and injection of all three vascular layers of the eye wall [1–3]. Prevalence estimates range from 3 to 8 per 100,000 person years [4–6]. Scleritis can be subdivided by time course into acute and chronic [1, 2]. It can also be categorized anatomically as anterior or posterior. Additional classification types include nodular versus diffuse; and necrotizing versus non-necrotizing. Among these various subtypes, acute diffuse non-necrotizing anterior scleritis is the most common. Anterior scleritis is idiopathic in approximately 50% of cases [1–5] but a thorough evaluation is necessary to rule out underlying systemic or localized causes. Treatment is multi-faceted with the treatment strategy potentially abled to be targeted to the underlying pathophysiology [7]. In this case report, we present an interesting presentation of chronic unilateral non-necrotizing nodular anterior scleritis associated with IgG4 lymphoplasmacytic inflammatory disease.

## Case report

A fifty-two-year-old female with a history of hypertension, diabetes mellitus, rheumatoid arthritis, ulcerative colitis, and recurrent episodes of pancreatitis presented for evaluation of a greater than one year history of intermittent recurring pain and redness of the right eye. The first episode also included severe photophobia but that was not an ongoing feature. Several episodes resolved without treatment. The patient had a remote history of radial keratotomy in both eyes and dacryocystorhinostomy of unknown laterality. Patient’s and family’s ocular history was otherwise unremarkable. She was taking adalimumab 40 mg subcutaneously every other week for her rheumatoid arthritis and ulcerative colitis.

Best corrected Snellen visual acuity at presentation was 20/30 + 2 in the right eye and 20/20 − 1 in the left eye. Intraocular pressure of right eye was elevated at 24 mmHg and normal in left eye. Pupils, confrontational visual fields, and extraocular motility were normal. On anterior segment exam, there was diffuse 2 + injection with nodularity in the right eye and 8 cut radial keratotomy in both eyes. Dilated fundus exam and macular optical coherence tomography (OCT) were normal in both eyes. The patient was diagnosed with acute unilateral non-necrotizing nodular anterior scleritis associated with rheumatoid arthritis and ulcerative colitis. Since she was already on systemic immunosuppression in the form of adalimumab, localized therapy was chosen and she was started on topical difluprednate one drop eight times daily in the right eye with improvement, followed by a 50% taper every seven days. The patient experienced another episode approximately 6–8 weeks after initial presentation which responded to a repeat course of topical difluprednate. Topical dorzolamide/timolol one drop twice daily was added due to increased intraocular pressure.

**Fig. 1:**
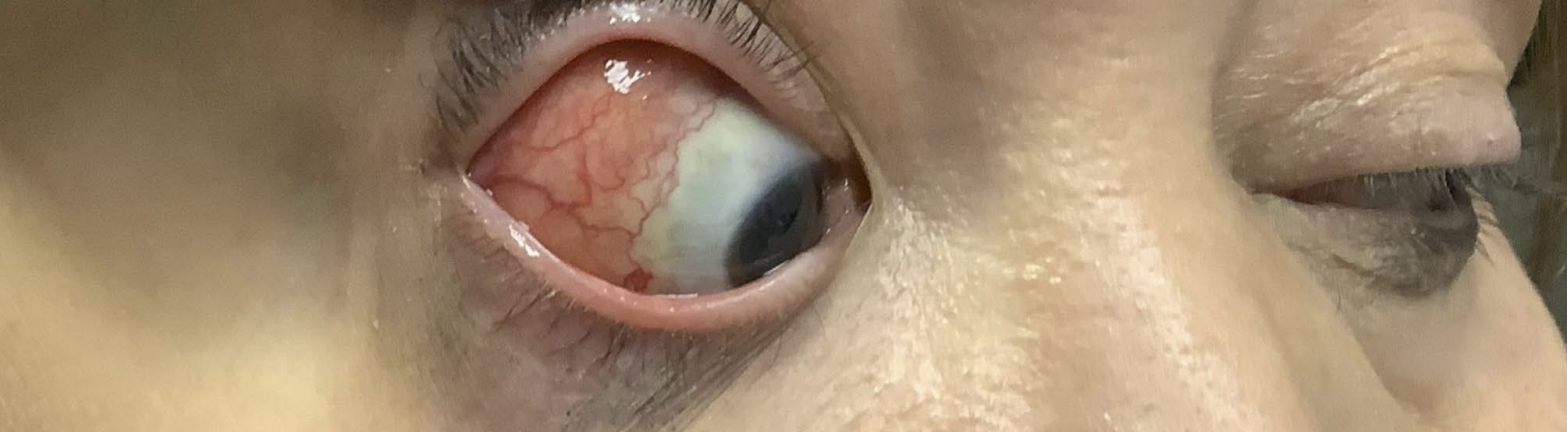
Color photo of anterior segment of right eye at 1 year

**Fig. 2:**
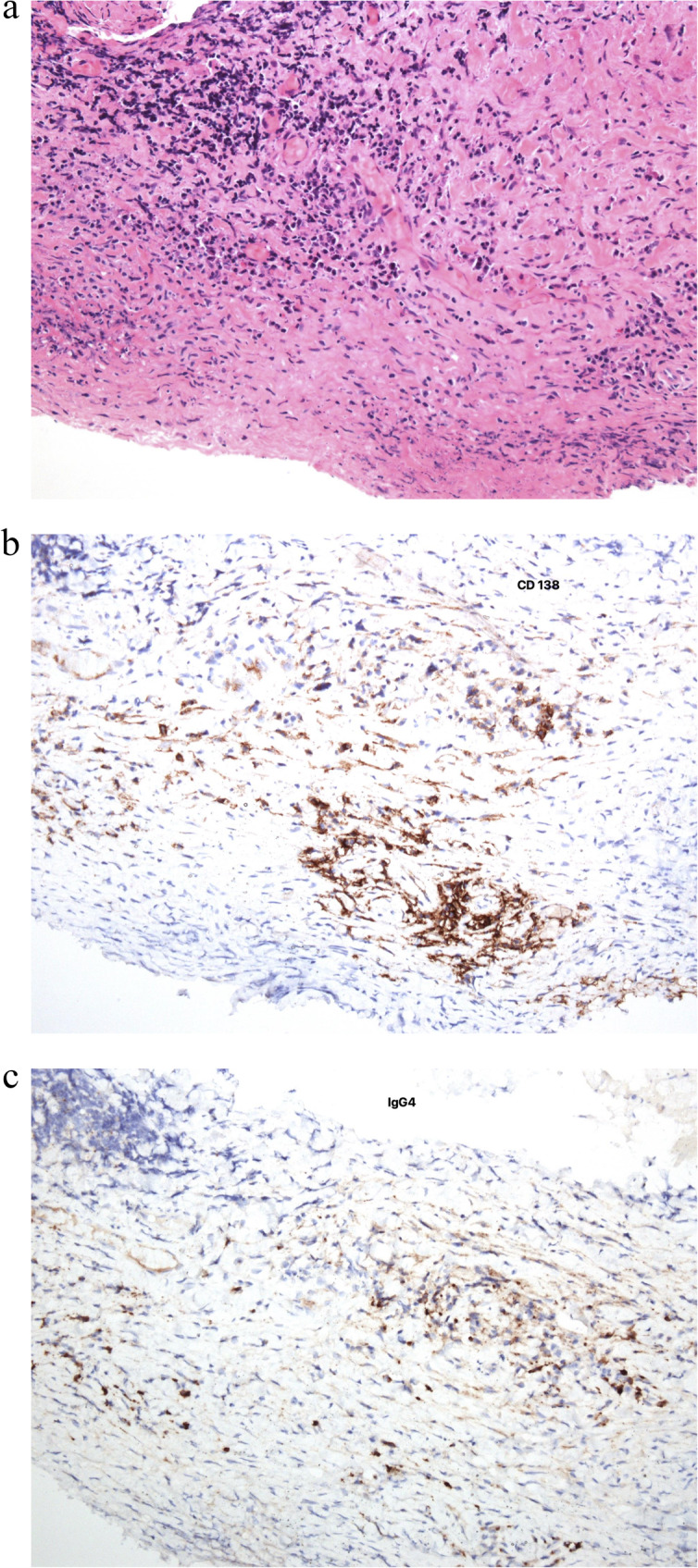
Histological findings of scleral biopsy of right eye **a** Hematoxylin-eosin stain at low-power field showing a marked lymphocytic plasmatic infiltrate. **b** Immunohistochemistry study demonstrating a diffuse pattern of IgG4-positive cells: a high proportion of the plasma cells are positive for IgG4 (~ 40%). **c** Immunohistochemistry study for CD138 plasma cell marker

The patient experienced recurrent symptoms one year after initial presentation. Vision, intraocular pressure, pupils, confrontational visual fields, and extraocular motility were normal. On anterior segment examination, there was sharply demarcated injection with overlying tortuous vessels in the right eye (Fig. 1). The anterior segment exam of the left eye, dilated fundus exam of both eyes and OCT macula of both eyes remained unremarkable. The atypical appearance with sharp delineation of vascular injection and nodular elevation raised concern for an infiltrative process and the patient was referred for orbital imaging and to the oculoplastic team for consideration of biopsy. Serum comprehensive metabolic panel, complete blood count with differential, and computed tomography (CT) of orbits was unremarkable. The patient underwent multiple superficial shave biopsies of the area of elevation. Serum immunoglobulin G (IgG) and IgG4 were within normal limits, although the patient was on systemic steroids (prednisone 60 mg daily) for anterior scleritis. The routine H&E stain of the biopsy specimen showed dense fibrosis with a lymphoplasmacytic infiltrate with a few eosinophils (Fig. 2a). The CD138 immunostain highlighted the plasma cells and an immunnostain for IgG4 marked the subset of plasma cells producing IgG4 (Fig. 2b). In the IgG4 positive focus there are greater than 10 positive cells/hpf confirming IgG4-related Ophthalmic Disease (Fig.2c). Following the biopsy, the patient was started on rituximab infusions and slowly tapered from the systemic prednisone and has been stable without recurrence of scleritis for 1 year.

## Discussion

The pathophysiology of scleritis involves inflammation of scleral and episcleral tissue. The proposed mechanisms vary widely based on the subtype of scleritis and associated systemic causes including infectious, autoimmune, depositional, and drug induced. Idiopathic scleritis without any known systemic conditions can account for up to 50% of patients [1–3]. This case highlights the importance of including IgG4-related disease (IgG4-RD) in the differential for underlying causes of scleritis.

A widely accepted diagnostic criteria for IgG4-related ophthalmic disease (IgG4-ROD) described by Goto el al [8] incorporates a combination of enlargement of orbital tissues on imaging, marked plasmacytic infiltration on histopathology and elevated serum IgG4. Our patient meets the criteria for possible IgG4-related orbital disease, emphasizing the importance of evaluating other aspects of clinical history including history of episodes of pancreatitis, idiopathic retroperitoneal or aortic fibrosis, renal, parotid gland, and lacrimal gland involvement [9–11]. Isolated inflammation of sclera is an uncommon presentation of IgG4-ROD. Typically, lacrimal gland, followed by extraocular muscles and orbital fat are more commonly affected [10, 11]. Interestingly, prior individual case reports and case series have highlighted IgG4 disease as a cause of scleritis, however few have reported scleritis as an isolated ophthalmic manifestation of IgG4-ROD [9, 12–14]. 

While the exact inciting factor of IgG4-ROD is still unknown, proposed mechanisms have included a local inflammatory cascade triggered by infectious pathogen, autoantigens, or genetic predisposition [10, 11]. Regardless of the spectrum of disease, the involved tissues show a lymphoplasmacytic infiltration leading to obliterative phlebitis and fibrosis if left untreated or undertreated [8, 10, 15]. The current case demonstrated a partial response to initial topical corticosteroid and systemic immunomodulatory therapy, highlighting the need for tailoring therapy to individual response as well as high likelihood of disease relapse [3, 9, 10]. 

## Conclusions

In summary, this case reports adds to the limited literature on scleritis as an isolated ophthalmic presentation of IgG4-RD. It is unknown at this time whether this presentation highlights a specific inciting or causal factor and therefore whether treatment regimen can be targeted for improved ocular and systemic IgG4-RD morbidity.

## Data Availability

No datasets were generated or analysed during the current study.

## Abbreviations

CT: Computed tomography
IgG: Immunoglobulin G
IgG4-RD: Immunoglobulin G4-related disease
IgG4-ROD: Immunoglobulin G4-related ophthalmic disease
OCT: Optical coherence tomography
